# High Genetic Diversity of Microbial Cellulase and Hemicellulase Genes in the Hindgut of *Holotrichia parallela* Larvae

**DOI:** 10.3390/ijms160716545

**Published:** 2015-07-21

**Authors:** Ping Sheng, Yushan Li, Sean D. G. Marshall, Hongyu Zhang

**Affiliations:** 1 State Key Laboratory of Agricultural Microbiology, Institute of Urban and Horticultural Pests, Hubei Insect Resources Utilization and Sustainable Pest Management Key Laboratory, College of Plant Science and Technology, Huazhong Agricultural University, Wuhan 430070, China; E-Mails: shengping_1014@163.com (P.S.); liyushan01@163.com (Y.L.); 2 Institute of Biological Resources, Jiangxi Academy of Sciences, Nanchang 330096, China; 3 Hubei Collaborative Innovation Center for the Characteristic Resources Exploitation of Dabie Mountains, Huanggang 438000, China; 4 Innovative Farming Systems, AgResearch, Lincoln Research Centre, Christchurch 8140, New Zealand; E-Mail: sean.marshall@agresearch.co.nz

**Keywords:** cellulase, hemicellulase, gene diversity, *Holotrichia parallela*

## Abstract

In this study, we used a culture-independent method based on library construction and sequencing to analyze the genetic diversity of the cellulase and hemicellulase genes of the bacterial community resident in the hindgut of *Holotrichia parallela* larvae. The results indicate that there is a large, diverse set of bacterial genes encoding lignocellulose hydrolysis enzymes in the hindgut of *H. parallela*. The total of 101 distinct gene fragments (similarity <95%) of glycosyl hydrolase families including GH2 (24 genes), GH8 (27 genes), GH10 (19 genes), GH11 (14 genes) and GH36 (17 genes) families was retrieved, and certain sequences of GH2 (10.61%), GH8 (3.33%), and GH11 (18.42%) families had <60% identities with known sequences in GenBank, indicating their novelty. Based on phylogenetic analysis, sequences from hemicellulase families were related to enzymes from Bacteroidetes and Firmicutes. Fragments from cellulase family were most associated with the phylum of Proteobacteria. Furthermore, a full-length endo-xylanase gene was obtained, and the enzyme exhibited activity over a broad range of pH levels. Our results indicate that there are large number of cellulolytic and xylanolytic bacteria in the hindgut of *H. parallela* larvae, and these symbiotic bacteria play an important role in the degradation of roots and other organic matter for the host insect.

## 1. Introduction

The family *Scarabaeidae* is currently defined as comprising over 30,000 species of beetles, which are almost exclusively herbivorous or saprophagous [1]. Many scarab larvae live in the soil and feed on peanut, plant roots and other organic matters [2]. In China, the phytophagous scarab *H. parallela* larva is a severe peanut and sweet potato crops pest [3]. The hindgut of scarab larvae is similar to that of the wooding-feed termite: It is enlarged and houses diverse microbes [2,4]. Previous studies have shown that 25%–65% of the ingested pure cellulose or neutral detergent fibers in scarab larvae’s diet are degraded by scarab larvae and their intestinal bacteria [4,5]. Furthermore, cellulolytic and hemicellulolytic bacteria have been isolated from the hindgut of some scarab species [6,7]. The scarab gut may be a potential source of bioreactor activities for bio-fuel production [1].

Lignocellulose is the major component of biomass in nature, and it can be utilized to produce ethanol. Hemicellulose, cellulose and lignin are its three main components, and they constitute 20%–40%, 40%–60%, and 10%–25% of lignocellulosic biomass, respectively [8]. Enzymatic hydrolysis of lignocellulosic materials is of great interest to various industries because the process can be environmentally friendly, high efficiency, and have lower energy requirements than physical and chemical hydrolysis [9]. Enzymes such as cellulase and hemicellulase are used in the hydrolysis of lignocellulosic materials in bioethanol production. Endoglucanases, belonging to glycosyl hydrolase (GH) family 8 (EC 3.2.1.4), and endo-xylanases, belonging to glycosyl hydrolase families 10 (EC 3.2.1.8) and 11 (EC 3.2.1.8), are the most abundant family of endoglucanases and endo-xylanases and have been extensively studied [10,11].

Two strategies are usually used to explore glycosyl hydrolase genes from complicated samples, such as soil, insect guts, rumen, and so on: metagenomic library construction followed by clone screening or culture-independent molecular method based on PCR. Wang *et al.* [12] explored the genetic diversity of endo-xylanases in goat rumen contents by using the culture-independent method, which indicated that the method could be used to analyze the functional gene diversity in some other ecosystems.

In this study, we report on a phylogenetic analysis of bacterial cellulase and hemicellulase genes cloned from the hindgut of *H. parallela* larvae using degenerate primers. The objective was to shed further light on the diversity of these genes in these lignocellulose-metabolizing ecosystems and to complement studies on bacterial lignocellulose degradation system in *H. parallela* larvae.

## 2. Results

### 2.1. Cellulase and Hemicellulase Gene Cloning and Diversity Analysis

PCR with degenerate primers using metagenomic DNA from *H. parallela* larvae hindgut content yielded amplicons (with size between 154 and 344 bp) for β-galactosidase (GH2), β-1,4-endoglucanase (GH8), endo-xylanase (GH10, 11), α-galactosidase (GH36) enzymes. However, genes from the other β-1,4-endoglucanase (GH5, 45), cellobiohydrolase (GH48), β-glucosidase (GH3), and β-xylosidase (GH39, 52) enzyme families were not found in this study.

**Figure 1:**
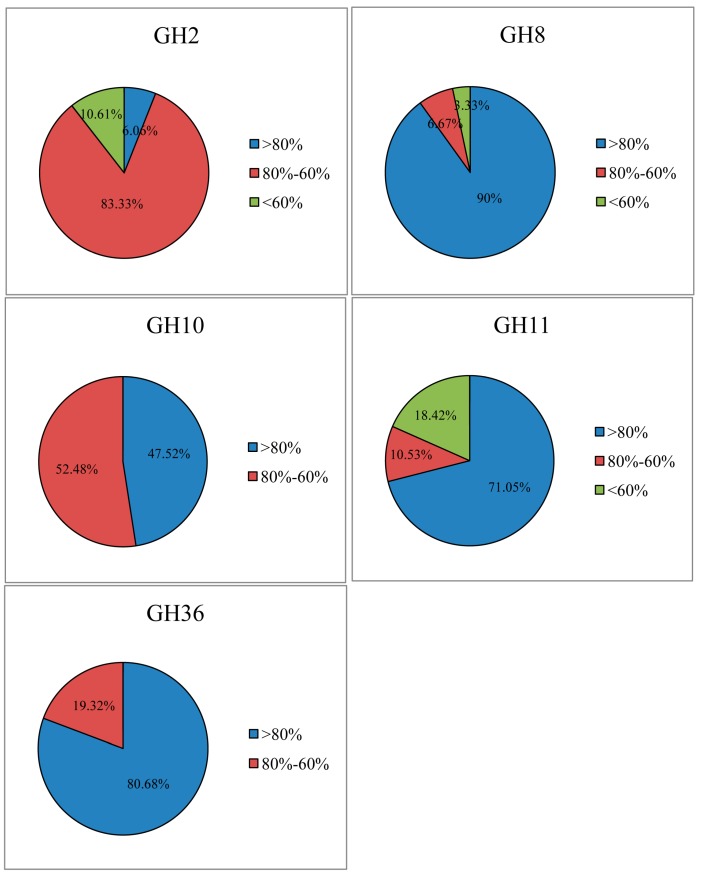
Amino acid sequence identities of cellulase and hemicellulase gene fragments to known enzymes. Each sequence was analyzed with NCBI BLASTp against the GenBank database.

**Figure 2:**
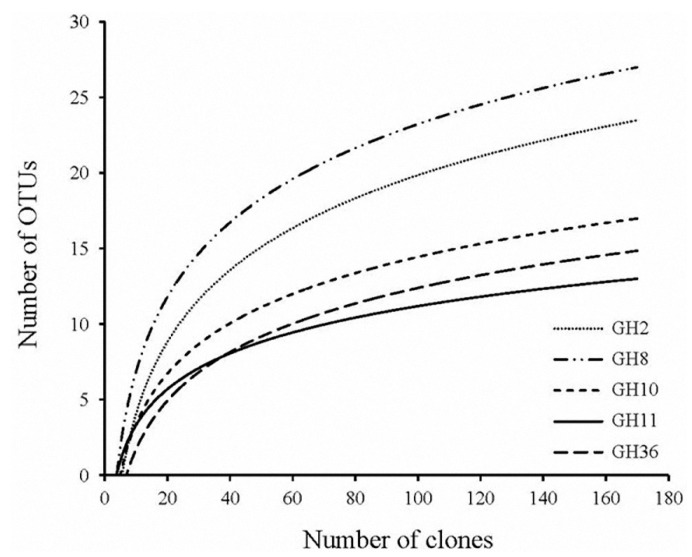
Rarefaction curves corresponding to the cellulase and hemicellulase genes from the hindgut of *Holotrichia parallela* larvae. Operational taxonomic units (OTUs) were defined at the 3% level of sequence divergence.

In the GH2, 8, 10, 11, and 36 families, 157, 170, 164, 160 and 170 clones showed 51%–99% amino acid identity with known microbial cellulases and hemicellulases based on BLAST analysis, and among them, 24, 27, 19, 14 and 17 clones showed sequence divergence (sharing <95% identity), respectively. Of these distinct fragments, 93.94%, 10%, 52.48%, 28.95% and 19.32% of GH2, GH8, GH10, GH11 and GH36 sequences were <80% identical with those of known GH enzymes, respectively. Moreover, 10.61%, 3.33% and 18.42% of GH2, GH8 and GH11 sequences had <60% identities with the available sequences in the NCBI database (Figure 1, Tables S1–S6). Therefore, it appears that a large number of cellulase and hemicellulase gene sequences discovered in this study originate from unknown micro-organisms. The rarefaction curves tended to approach the saturation plateau, indicating a sufficient sampling depth of all libraries (Figure 2).

**Figure 3:**
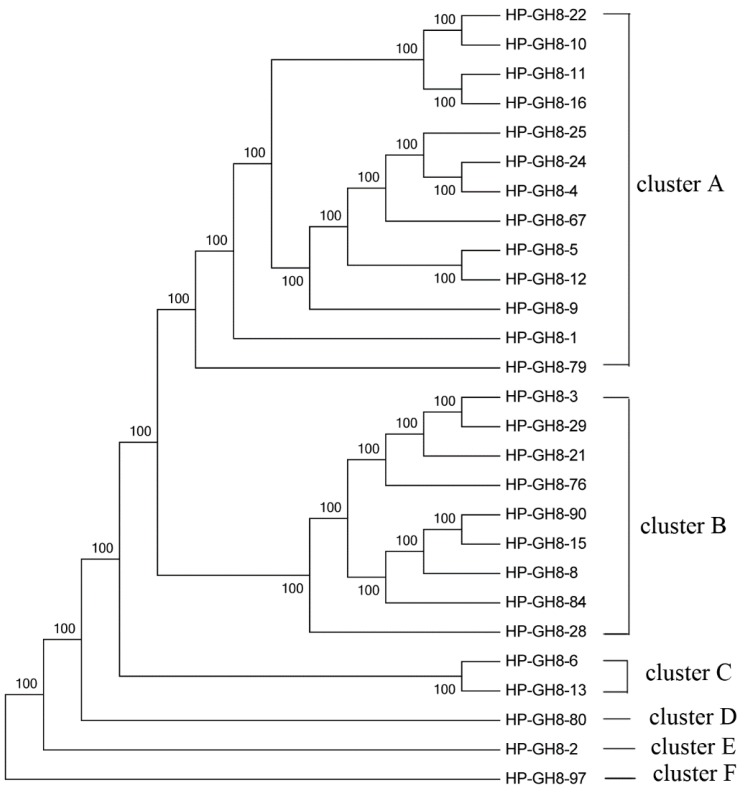
Maximum Likelihood phylogenetic tree of the GH8 amino acid sequences.

### 2.2. Phylogenetic Analysis of GH8 Endoglucanase Gene Fragments

The phylogenetic tree of the GH8 (β-1,4-endoglucanase) family was constructed using 27 distinct sequences. These sequences were devided into six clusters (Figure 3), indicating substantial diversity among GH8 endoglucanases in the scarab larval hindgut. From Figure 3, we found that most sequences grouped together except GH8-80, 2, and 97. Interestingly, 25 sequences were closed related to endoglucanases of Proteobacteria, and most of them belonged to the *Enterobacter*-like endoglucanase. The other two sequences were related to enzymes of Actinobacteria (Table S2). Our results indicated there is a rich genetic diversity of cellulolytic Proteobacteria species in the hindgut of *H. parallela* larvae.

### 2.3. Phylogenetic Analysis of GH2 and GH36 Galactosidase Gene Fragments

**Figure 4:**
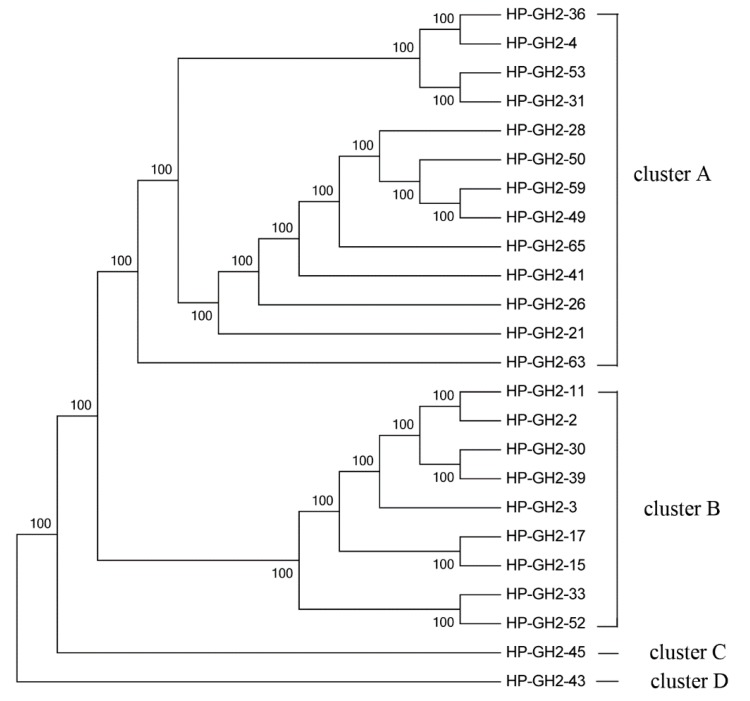
Maximum Likelihood phylogenetic tree of the GH2 amino acid sequences.

The phylogenetic tree of the GH2 family was constructed using 24 distinct sequences. These sequences were devided into four clusters (Figure 4). They were closed related to galactosidase of Bacteroidetes, Firmicutes, Proteobacteria, Arthropoda, Planctomycetes and some other uncultured bacteria (Table S3).

**Figure 5:**
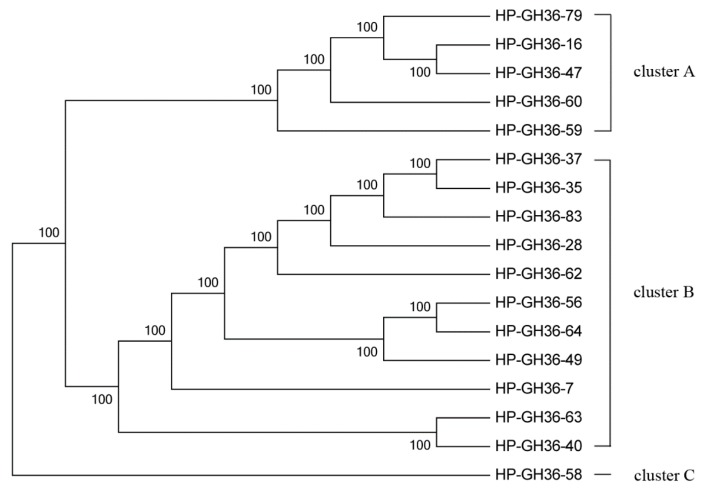
Maximum Likelihood phylogenetic tree of the GH36 amino acid sequences.

A total of 17 sequences from the GH36 α-galactosidase enzyme family were used to construct the phylogenetic tree. Three clusters were separated in this tree (Figure 5). Among these sequences, eight sequences were closely related to galactosidases of Bacteroidetes, most of them belonged to the *Dysgonomonas*-like galactosidases. Seven sequences were closely related to galactosidases of Firmicutes, only few sequences were related to enzymes of Acidobacteria and Proteobacteria (Table S4). These results indicate the high diversity of enzymes from Bacteroidetes and Firmicutes species in the scarab larval hindgut.

**Figure 6:**
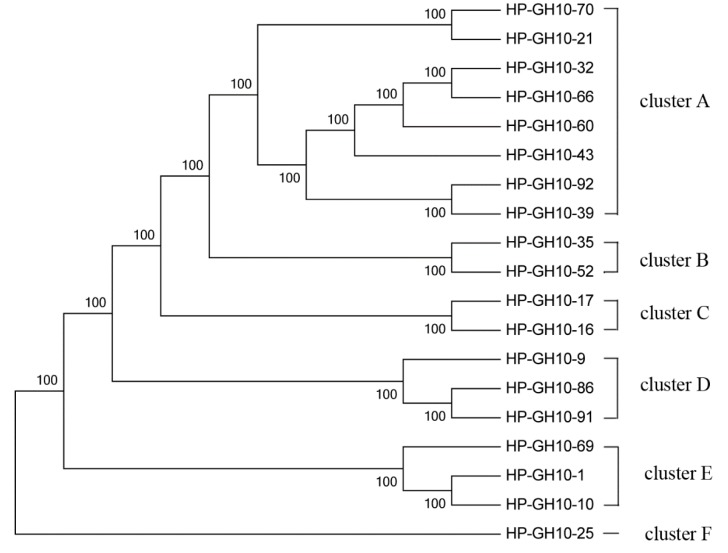
Maximum Likelihood phylogenetic tree of the GH10 amino acid sequences.

### 2.4. Phylogenetic Analysis of GH10 and GH11 Endo-Xylanase Gene Fragments

The 19 distinct partial sequences of GH10 endo-xylanase were used to construct a phylogenetic tree. The sequences from GH10 families were confined to six clusters (Figure 6). Most of them were closely related to endo-xylanases of Bacteroidetes, Firmicutes and Proteobacteria. Others were related to enzymes of uncultured bacteria (Table S5).

**Figure 7:**
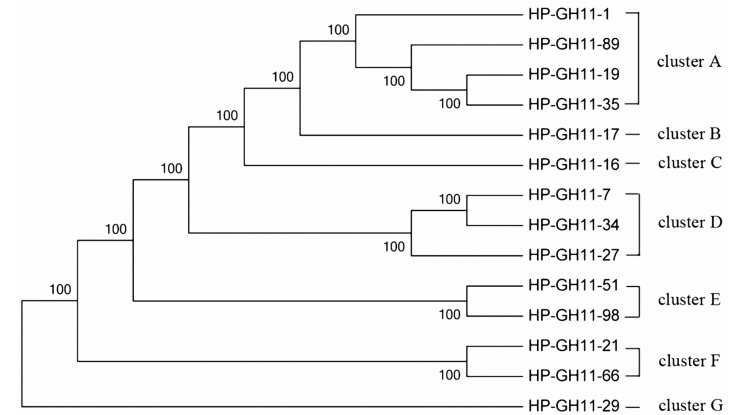
Maximum Likelihood phylogenetic tree of the GH11 amino acid sequences.

The phylogenetic tree of the GH11 family was constructed using 14 distinct sequences. These sequences were divided into seven clusters (Figure 7), indicating substantial diversity among GH11 endo-xylanases in the scarab larval hindgut. They were closely related to endo-xylanases of Firmicutes, Bacteroidetes, and Ascomycota. Among them, many sequences belonged to the *Dysgonomonas*-like endo-xylanases (Table S6).

### 2.5. Gene Cloning, Expression, and Characterization of Endo-Xylanase

The partial endo-xylanase sequence GH11-7, which showed the lowest identity (53%) with known sequences, was selected for further study. The full-length xynGH11-7 endo-xylanase gene was directly cloned from the metagenomic DNA from the *H. parallela* larval hindgut. The ORF for xynGH11-7 is 711 bp, encoding 237 amino acids. The protein is predicted have a molecular mass of 26.7 kDa with no signal peptide. The predicted theoretical pI for xynGH11-7 was at pH 6.07, and it is predicted to be hydrophilic. Sequence analysis of the amplified xynGH11-7 fragment exhibited the highest amino acid sequence identity (50%) to an endo-xylanase gene in the GH 11 family from *Bacillus* sp. NCL (Figure S1).

**Figure 8:**
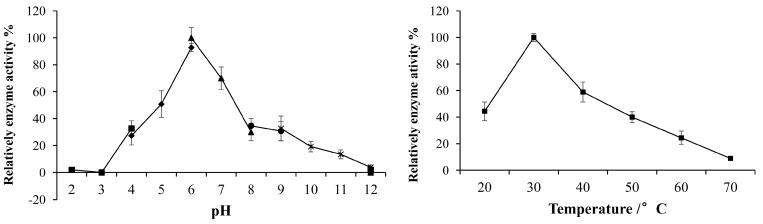
Optimal pH and temperature for the activities of recombinant xynGH11-7.

**Figure 9:**
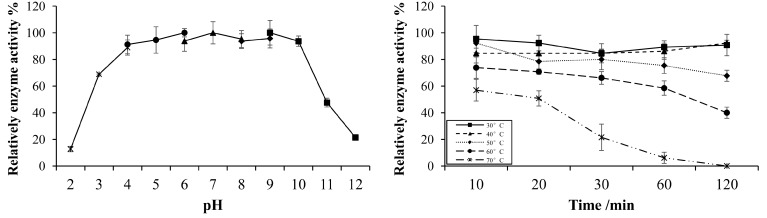
Stability of recombinant xynGH11-7 at different pH and temperatures.

The optimal pH and temperature for xynGH11-7 enzyme activities on xylan were observed at pH 6.0 and 30 °C, respectively (Figure 8). Furthermore, we found that the purified recombinant xynGH11-7 was stable at a pH range of 4.0 to 10.0, and approximately 88%–93% of the original enzymatic activity was still maintained after 1 h incubation at various pH levels (Figure 9). The thermal stability study shown in Figure 8 revealed that the enzyme was stable from 30 to 50 °C for approximately 2 h incubations, but was dramatically inactivated when temperatures reached 60 °C.

## 3. Discussion

In this study, cellulase and hemicellulase genes originating from symbiotic bacteria residing in the phytophagous *H. parallela* larval hindgut were targeted for diversity analysis because of their synergistic roles in lignocelluloses degradation. Using culture-independent methods, 101 different endo-xylanase, galactosidase, and β-1,4-endoglucanase sequences were isolated from the *H. parallela* larval hindgut. The results are consistent with previous studies of gut bacterial communities in *H. parallela* larvae, in which rich collections of cellulolytic and xylanolytic bacteria were found in the hindgut of *H. parallela* larvae [1,2,6,7]. Similar phenomena have also been observed in the wood-feeding higher termite [13] and another scarab beetle, *Pachnoda marginata* [14]. A common feature of these insects is that they all eat high-fiber diets, and that likely explains why large, diverse sets of bacterial genes for cellulose and xylan hydrolysis were found in their hindguts. Recent data also support the idea that the gut symbiotic bacteria are important in lignocellulose hydrolysis for the host insects [13,15,16].

Enzyme fragments from GH2, 8, 10, 11, and 36 families were obtained, suggesting that the gut of *H. parallela* larvae harbor diverse cellulolytic and xylanolytic microbes and enzymes. β-Galactosidases and α-galactosidases from GH2 and GH36 are known to degrade β-d-galactosides and α-d-galactosides, respectively. Endoglucanases from GH8 can degrade cellulose, and endo-xylanases from GH10 and GH11 were reported to degrade xylan (http://www.cazy.org/Glycoside-Hydrolases.html). Furthermore, we did not find sequences from the GH3, 5, 39, 45, 48, and 52 enzyme families. A similar phenomenon was also observed in the termite hindgut, where a pyrosequenced hindgut microbiota metagenome analysis by Warnecke *et al*. showed a smaller number of GH39, GH45, and GH52 enzyme sequences, with gene fragments from the GH48 family cellobiohydrolase being completely absent [13].

Proteobacteria is the most abundant bacterial phylum in the hindgut of *H. parallela* larvae, and Actinobacteria is the second most abundant [6]. A 16S rDNA analysis of the hindgut microbiota of *H. parallela* larvae from different geographic regions by Huang *et al*. also showed that the Enterobacteriaceae family of the Proteobacteria phylum was commonly found in all of the natural populations, and constituted dominant and stable populations in the scarab gut [17]. Our current study showed that 25 distinct sequences from the GH8 β-1,4-endoglucanase family were closely related to this enzyme from Proteobacteria, comprising 92.59% (25/27) of the total distinct sequences, and the remaining sequences were related to enzymes from Actinobacteria (Table S2). Among them, 85.19% (23/27) of the total distinct sequences belonged to the family Enterobacteriaceae (Table S2). Consulting the collection of GH8 bacterial sequences in the CAZy database (http://www.cazy.org/), we also found that the phylum of Proteobacteria predominated, followed by Firmicutes. A similar result has also been reported by *Elifantz* (2008) in a study of endoglucanase diversity in marine bacteria [18]. Moreover, members of the Enterobacteriaceae family have been implicated in nitrogen and carbon metabolism in the fruit fly *Ceratitis capitata*, and they have an indirect contribution to host fitness [19]. Our results suggest a possible cellulose degradation function for these bacterial species in the host insect. However, the main endoglucanase enzyme in wood-feeding “higher” Nasutitermes hindgut was attributed to the GH5 family [13]; in the *H. parallela* larvae hindgut, it was assigned to the GH8 family, and no enzyme sequences belonging to the GH5 family were cloned. The species differences observed in cellulose degradation enzymes may be due to host insect differences, including diet [20,21].

Based on phylogenetic analysis, the GH10 and GH11 endo-xylanase sequences were closely related to the endo-xylanases from Bacteroides and Firmicutes (Tables S5 and S6). These results are similar to those of previous studies. Based on the collected endo-xylanase sequences in the Pfam database, Bacteroidetes and Firmicutes are the main microbial source of endo-xylanase production. Interestingly, in goat and sheep rumens and in some other herbivores (e.g., wallabies), endo-xylanase genes were also found to be mainly distributed between these two phyla [12,19]. However, the distribution of GH10 and GH11 endo-xylanases in the scarab hindgut differed from both that of tundra soil (in which bacterial endo-xylanase genes were mainly detected from Actinobacteria and Proteobacteria) [22] and the termite hindgut (in which endo-xylanases were mainly from the phyla of Fibrobacteres and Spirochaetes) [13]. Additionally, in comparison with endo-xylanase sequences of the GH11 family, the GH10 family possessed much more gene richness. This result was similar to that found in other microenvironments, such as the termite hindgut [13], and goat and cow rumen [12,23]. GH11 endo-xylanases have a lower catalytic versatility than GH10 endo-xylanases, and the products of their action can be further hydrolyzed by the GH10 enzymes [24,25]. Therefore, the difference in gene richnesses between these two families implies that they may have different roles in xylan degradation in the hindgut.

Furthermore, we cloned, expressed and biochemically characterized one endo-xylanase. To our knowledge, no GH 11 xylanase has been isolated from the hindgut of *H. parallela* larvae before. The enzyme showed a broad pH stability, indicating that this enzyme is promising for applications in many industries, such as the paper industry, which requires enzymes to exhibit pH stability over a wide range [26]. Some xylanases had also been cloned from the gut of other insects, such as *Gryllotalpa orientalis* [27,28], *Batocera horsfieldi* larvae [29], however, most of them showed a narrower pH stability. Another prominent feature of the xynGH11-7 enzyme is its low identity (50%) with known endo-xylanases in the NCBI database, further suggesting the novelty of this enzyme.

In conclusion, large numbers of cellulase (endoglucanase) and hemicellulase (galactosidase and endo-xylanase) fragments from the GH2, GH8, GH10, GH11 and GH36 families were cloned from the hindgut of *H. parallela* larvae using culture-independent molecular methods. Some of the sequences have low identities with known glycosyl hydrolases, suggesting the phylogenetic diversity and novelty of these enzyme genes. Enzyme fragments amplified in our work were mostly distributed to the bacterial species in Proteobacteria, Bacteroides and Firmicutes, which indicate potential contributors to lignocellulose degradation in the *H. parallela* larval hindgut. Full length cloning and heterologous expression of an endo-xylanase-like gene further indicated its function as an active endo-xylanase. Taken in combination with previous studies on *H. parallela* larvae, our work highlights the hindgut of the larvae as a reservoir of extensive and specific cellulase and hemicellulase sequence diversities.

## 4. Materials and Methods

### 4.1. Ethics Statement

*H. parallela* has not been notified under any act or laws and rules thereof of the Government of China as an endangered or threatened species restricting or regulating its collection and observation. The peanut field was privately owned, and we have the land owner’s consent to access the land for collecting the larvae, there are no permits required for the land use or collection of the larvae.

### 4.2. Insect Samples

Healthy early third-instar grub larvae were collected from a peanut field (E: 112.199266°, N: 31.035516°) and then reared in a terrarium in the lab. The terrarium was filled with organic soil, held at a constant temperature of 27 ± 1 °C and light-dark photoperiod of 14:10 h, as described by Huang *et al.* [17]. Larvae were fed with peanut roots until they were used. Before dissection, larvae were separated and reared in a sterile container without food and soil for 2 days.

### 4.3. Insect Dissection and Total Bacterial DNA Extraction

Insect dissections were performed according to the method described by Zhang and Jackon [2]. Briefly, grub larvae were surface-sterilized with 75% ethanol and then washed in sterile distilled water. All further steps were performed under sterile conditions. Fifty entire hindguts were removed from abdomens and then homogenized. Total bacterial genomic DNA was extracted using a QIAamp stool DNA extraction kit (Qiagen, Hilden, Germany), and the genomic DNA was stored at −70 °C before use.

### 4.4. PCR Amplification, Library Construction and Sequencing

The degenerate primers used for amplification of cellulase and hemicellulase genes are listed in Table 1, they are according to the method described by Wang *et al*. [22]. The purified bacterial genomic DNA was used as a template and underwent a touchdown PCR. The PCR was performed using a thermal cycler in a 50 μL reaction mix. The detail amplification procedures for the targeted gene families are shown in Table 2. The PCR-amplified gene products were cloned using the TA cloning method of the pEASY-T1 cloning kit (Transgen, Beijing, China) according to the manufacturer’s instructions for sequencing. Clones from each enzyme family were selected for sequencing, which was conducted by Sangon Biotech (Shanghai) Co., Ltd.

**Table 1:** Degenerate primers designed to amplify cellulase and hemicellulase gene fragments.

Pfam Family	Enzyme	EC Number	Primer (5ʹ–3ʹ)	Length bp
GH2	β-Galactosidase	EC3.2.1.23	GH2F: GTGCGYACSWSBCAYTAYCC	204–219
			GH2R: CCAAATRAYRAYGCTYGGRTGRTT	
GH3	β-Glucosidase	EC3.2.1.21	GH3F: GTKAAYCCRWSYGGIMRIYT	183–200
			GH3R: TAISWYAKICCRTRVCCRAA	
GH5	β-1,4-Endoglucanase	EC3.2.1.4	GH5F: TWYGARYTIYTIAAYGARC	195–258
			GH5R: NGGRTTRTARWARTGRAA	
GH8	β-1,4-Endoglucanase	EC3.2.1.4	GH8F: GAAGGYCWGGGYTWYGSVATG	183–207
			GH8R: AATMWSYWSATCRCCATCGSTSGC	
GH10	Endo-xylanase	EC3.2.1.8	GH10F: GGYCAYACBCTNRTNTGGCA	138–186
			GH10R: YTCRTTNACNACRTCCCA	
GH11	Endo-xylanase	EC3.2.1.8	GH11F: TAYMTGDSNSTBTAYGGBTGG	336
			GH11R: TRCCVCTVCTYTKRTAVCCYTC	
GH36	α-Galactosidase	EC3.2.1.22	GH36F: GACATGTTCGTGATGGACGAYGGNTGGTT	193
			GH36R: CGGACTCTGGGTTCACCATYTCNGGYTC	
GH39	β-Xylosidase	EC3.2.1.37	GH39F: TTYGARGTNTGGAAYGARCC	223–230
			GH39R: GCRTGNCKISWIACRAARTC	
GH45	β-1,4-Endoglucanase	EC3.2.1.4	GH45F: ACCMGITAYTGGGAYTGYTG	377–413
			GH45R: AAGRYICCNAVICCNCCICCNGG	
GH48	Cellobio-Hydrolase	EC3.2.1.91	GH48F: GARGCNCCNGAYYAYGGICA	420
			GH48R: CCNCGYTGRWAIGTRTTDA	
GH52	β-Xylosidase	EC3.2.1.37	GH52F: GARGGNGARTAYMGIATGATGAAYAC	197–200
			GH52R: GCVACNCCCATRTCRTGNGT	

**Table 2:** Settings for amplification of cellulase and hemicellulase gene fragments.

Standard	Touchdown PCR Settings	
	94 °C 5 min	
	94 °C 30 s, *X* °C 30 s (−0.5 °C/cycle), 72 °C 30 s; 30 cycles	
	94 °C 30 s, *Y* °C 30 s, 72 °C 30 s; 20 cycles	
	72 °C 10 min	
	4 °C infinity	
**Pfam Family**	The following PCR settings are same as the Standard:	
GH2 (PF02836)	*X* = 62	*Y* = 52
GH3 (PF01915)	*X* = 65	*Y* = 50
GH5 (PF00150)	*X* = 58	*Y* = 52
GH8 (PF01270)	*X* = 65	*Y* = 55
GH10 (PF00331)	*X* = 60	*Y* = 48
GH11 (PF00457)	*X* = 60	*Y* = 48
GH36 (PF02065)	*X* = 65	*Y* = 58
GH39 (PF01229)	*X* = 65	*Y* = 50
GH45 (PF02015)	*X* = 68	*Y* = 55
GH48 (PF02011)	*X* = 65	*Y* = 50
GH52 (PF03512)	*X* = 68	*Y* = 57

### 4.5. Phylogenetic Analysis of Cellulase and Hemicellulase Gene Sequences

The cellulase and hemicellulase nucleotide sequences were translated using ExPASy tool (http://www.expasy.ch/tools/). All the deduced amino-acid sequences were aligned with ClustalX and the redundant sequences were removed with CD-HIT, using a sequence identity cutoff of 95% [30]. Gene abundance and rarefaction curves of each GH family was estimated using distance-based operational taxonomic unit and richness determination (DOTUR) software [31]. The coverage of the clone library was considered to be: *C* = 1 − (*n*1/*N*), where *n*1 is the number of single clones, and *N* is the total number of clones. All richness estimations and diversity indices were computed using EstimateS software (http://viceroy.eeb.uconn.edu/EstimateS). Phylogenetic analyses were conducted with the Maximum Likelihood (ML) method using the PHYLIP program [32].

### 4.6. Gene Cloning, Expression, and Characterization of Endo-Xylanase

Fragment xynGH11-7 was chosen for full-length gene cloning based on its low similarity (53%) to known endo-xylanase enzymes, which suggested potential for novelty, and was therefore chosen for full-length gene cloning. The full-length gene was obtained by thermal asymmetric interlaced-PCR (TAIL-PCR) using six nested, insertion-specific primers. The pPIC9K expression vector and *Pichia pastoris* were selected for enzyme expression, with the His_6_ tagged protein purified by chelation to a sepharose (Ni-NTA) resin matrix (Novagen, Merck, Kenilworth, NJ, USA). The details regarding oligonucleotide primers used for xynGH11-7 cloning and expression are provided in Table S1.

Enzyme activity was determined as described by Zhou [29], with several modifications: The release of reducing sugar from beech wood xylan was measured (1% *w*/*v* in acetate buffer, pH 6.0) at 37 °C for 10 min using a 3,5-dinitrosalicylic acid (DNSA) reagent. One unit (U) of endo-xylanase activity was defined as the amount of enzyme required to release 1 μmol of reducing sugars as xylose from xylan per minute under the reaction conditions.

The optimal pH was determined in a series of buffers with pH values ranging from 2.0 to 12.0, while the optimal temperature was determined over 10 °C intervals ranging from 20–70 °C. The buffers used were glycine-HCl (pH 2.0 to 3.0), acetate sodium-acetic acid (pH 4.0 to 6.0), dibasic sodium phosphate/sodium biphosphate (pH 6.0 to 8.0), and glycine-NaOH (pH 9.0 to 12.0). The pH stability of the enzyme activity was tested by pre-incubating the recombinant enzyme for 1 h at 37 °C and under pH conditions ranging from 2.0 to 12.0. The thermostability of the enzyme was determined by pre-incubating the enzyme for 1 h at 30–70 °C at a 10 °C intervals. The residual enzyme activities were assayed as described above.

### 4.7. Nucleotide Sequence Accession Numbers

The nucleotide sequences of the GH2, GH8, GH10, GH11 and GH36 gene fragments were deposited into the GenBank and DDBJ databases under accession numbers KF844174-KF844194, AB872357-AB872359, AB872360-AB872386, AB872387-AB872404, KF844195-KF844206, and AB872405-AB872421. The full-length endo-xylanase gene sequence has been submitted to the Genbank database with the accession number KF927127.

## Supplementary Materials

Supplementary materials can be found at http://www.mdpi.com/1422-0067/16/07/16545/s1.
