# Supplementary material for: High Genetic Diversity of Microbial Cellulase and Hemicellulase Genes in the Hindgut of Holotrichia parallela Larvae
Source: Int J Mol Sci. 2015 Jul 21;16(7):16545–59. doi: 10.3390/ijms160716545 (PMC4519965; doi:10.3390/ijms160716545)
Supplement: Supplementary file 1 [file ijms-16-16545-s001.pdf]

Supplementary Information

Table S1. Oligonucleotide primers used for xynGH11-7 cloning and expression.

Primer Name	Primer Sequence (5′–3′)	Size (Bases)
xynGH11-7-F	TAYMTGDSNSTBTAYGGBTGG	21
xynGH11-7-R	TRCCVCTVCTYTKRTAVCCYTC	22
xynGH11-7-uSP1	TATTGACCGAAGTCACGGACGCCAA	25
xynGH11-7-uSP2	TGCCGCCATCAGTATCATGTGCTCC	25
xynGH11-7-uSP3	ACGGATCTACCGTCCACCCATACAG	25
xynGH11-7-dSP1	AGGAGCACATGATACTGATGGCGGC	25
xynGH11-7-dSP2	GGGTCGTCTATCCTTGGCGTCCGTG	25
xynGH11-7-dSP3	AACATGGGTCTTACATTGGGCAACC	25
exynGH11-7-F	*GAATTC*ATGACACCGCTTCAATAC	24
exynGH11-7-R	*GCGGCCGC*ATGATGATGATGATGATGTTTTCATCTATTTCCT	43

Table S2. The group of GH 8 representative sequences identities.

Sequences	Closest Relative	Phylum	Number of Sequences	Identity %
HP-GH8-3	*Agrobacterium tumefaciens*	Proteobacteria	1	97
HP-GH8-76	*Cedecea davisae*	Proteobacteria	1	73
HP-GH8-8	*Cellulomonas uda CB4*	Actinobacteria	1	94
HP-GH8-15	*Cellulomonas uda CB4*	Actinobacteria	1	92
HP-GH8-16	*Enterobacter cloacae EC_38VIM1*	Proteobacteria	2	83
HP-GH8-4	*Enterobacter cloacae str. Hanford*	Proteobacteria	1	90
HP-GH8-24	*Enterobacter cloacae str. Hanford*	Proteobacteria	19	89
HP-GH8-25	*Enterobacter cloacae str. Hanford*	Proteobacteria	1	89
HP-GH8-1	*Enterobacter mori*	Proteobacteria	1	95
HP-GH8-2	*Enterobacter mori*	Proteobacteria	1	92
HP-GH8-5	*Enterobacter mori*	Proteobacteria	2	90
HP-GH8-6	*Enterobacter mori*	Proteobacteria	13	94
HP-GH8-9	*Enterobacter mori*	Proteobacteria	11	94
HP-GH8-10	*Enterobacter mori*	Proteobacteria	1	94
HP-GH8-12	*Enterobacter mori*	Proteobacteria	1	86
HP-GH8-13	*Enterobacter mori*	Proteobacteria	14	95
HP-GH8-97	*Enterobacter mori*	Proteobacteria	21	97
HP-GH8-79	*Enterobacter mori*	Proteobacteria	1	92
HP-GH8-20	*Enterobacter mori*	Proteobacteria	1	94
HP-GH8-22	*Enterobacter mori*	Proteobacteria	1	92
HP-GH8-67	*Enterobacter mori*	Proteobacteria	13	92
HP-GH8-28	*Enterobacter mori*	Proteobacteria	1	92
HP-GH8-11	*Enterobacter* sp. SST3	Proteobacteria	57	90
HP-GH8-21	*Escherichia hermannii*	Proteobacteria	1	76
HP-GH8-90	*Klebsiella oxytoca*	Proteobacteria	1	92
HP-GH8-84	*Klebsiella pneumoniae*	Proteobacteria	1	86
HP-GH8-29	*Sutterella* sp. CAG 521	Proteobacteria	1	52

Table S3. The group of GH 2 representative sequences identities.

Sequences	Closest Relative	Phylum	Number of Sequences	Identity %
HP-GH2-65	*Alistipes senegalensis*	Bacteroidetes	13	57
HP-GH2-3	*Bacillus megaterium WSH-002*	Firmicutes	1	78
HP-GH2-39	*Cellulosilyticum ruminicola*	Proteobacteria	16	72
HP-GH2-52	*Epulopiscium sp. 'N.t. morphotype B'*	Firmicutes	1	73
HP-GH2-4	*Flexithrix dorotheae*	Bacteroidetes	1	68
HP-GH2-21	*Flexithrix dorotheae*	Bacteroidetes	12	71
HP-GH2-36	*Flexithrix dorotheae*	Bacteroidetes	13	69
HP-GH2-41	*Flexithrix dorotheae*	Bacteroidetes	13	59
HP-GH2-28	*Formosa agariphila KMM 3901*	Bacteroidetes	25	68
HP-GH2-49	*Formosa agariphila KMM 3901*	Bacteroidetes	12	68
HP-GH2-59	*Formosa agariphila KMM 3901*	Bacteroidetes	1	68
HP-GH2-17	*Mucilaginibacter paludis*	Bacteroidetes	1	53
HP-GH2-26	*Mucilaginibacter paludis*	Bacteroidetes	1	63
HP-GH2-31	*Paenibacillus ginsengihumi*	Firmicutes	1	67
HP-GH2-2	*Paenibacillus* sp. JDR-2	Firmicutes	1	71
HP-GH2-11	*Paenibacillus* sp. JDR-2	Firmicutes	12	69
HP-GH2-63	*Pediculus humanus corporis*	Arthropoda	1	91
HP-GH2-43	*Rhodopirellula sallentina*	Planctomycetes	11	69
HP-GH2-45	*Rhodopirellula sallentina*	Planctomycetes	1	68
HP-GH2-50	*Sphingobacterium* sp. 21	Bacteroidetes	10	63
HP-GH2-15	*Tannerella* sp. CAG 118	Bacteroidetes	1	97
HP-GH2-30	*Turicibacter* sp. HGF1	Firmicutes	1	71
HP-GH2-33	uncultured bacterium	-	7	96
HP-GH2-53	uncultured bacterium	-	1	63

Table S4. The group of GH 36 representative sequences identities.

Sequences	Closest Relative	Phylum	Number of Sequences	Identity %
HP-GH36-56	*Acidobacteriaceae bacterium KBS 83*	Acidobacteria	1	79
HP-GH36-7	*Bacillus acidiproducens*	Firmicutes	17	72
HP-GH36-63	*Bacteroides* sp. HPS0048	Bacteroidetes	1	83
HP-GH36-35	*Clostridium phytofermentans ISDg*	Firmicutes	1	69
HP-GH36-83	*Clostridium phytofermentans ISDg*	Firmicutes	1	67
HP-GH36-49	*Coprococcus eutactus CAG 665*	Firmicutes	1	77
HP-GH36-16	*Dysgonomonas gadei*	Bacteroidetes	1	86
HP-GH36-40	*Dysgonomonas gadei*	Bacteroidetes	18	72
HP-GH36-47	*Dysgonomonas gadei*	Bacteroidetes	1	81
HP-GH36-58	*Dysgonomonas gadei*	Bacteroidetes	20	89
HP-GH36-59	*Dysgonomonas gadei*	Bacteroidetes	1	84
HP-GH36-60	*Dysgonomonas gadei*	Bacteroidetes	1	84
HP-GH36-79	*Dysgonomonas gadei*	Bacteroidetes	74	86
HP-GH36-62	*Lachnospiraceae bacterium A4*	Firmicutes	15	75
HP-GH36-28	*Roseburia hominis A2-183*	Firmicutes	1	64
HP-GH36-37	*Streptococcus agalactiae*	Firmicutes	1	67
HP-GH36-64	*Vibrio sinaloensis*	Proteobacteria	15	66

Table S5. The group of GH 10 representative sequences identities.

Sequences	Closest Relative	Phylum	Number of Sequences	Identity %
HP-GH10-69	*Agrobacterium fabrum str. C58*	Proteobacteria	1	82
HP-GH10-35	*Bacteroides* sp. CAG 875	Bacteroidetes	1	71
HP-GH10-52	*Bacteroides* sp. CAG 875	Bacteroidetes	27	71
HP-GH10-1	*Bacteroides xylanisolvens*	Bacteroidetes	40	87
HP-GH10-25	*Bacteroides xylanisolvens*	Bacteroidetes	1	82
HP-GH10-10	*Bacteroides*	Bacteroidetes	12	85
HP-GH10-16	*Chryseobacterium gleum*	Bacteroidetes	12	85
HP-GH10-17	*Chryseobacterium gleum*	Bacteroidetes	13	84
HP-GH10-39	*Clostridium phytofermentans ISDg*	Firmicutes	1	75
HP-GH10-21	*Dysgonomonas gadei*	Bacteroidetes	1	73
HP-GH10-32	*Paenibacillus curdlanolyticus*	Firmicutes	18	69
HP-GH10-60	*Paenibacillus curdlanolyticus*	Firmicutes	1	67
HP-GH10-66	*Paenibacillus curdlanolyticus*	Firmicutes	1	66
HP-GH10-91	*Paenibacillus* sp. JDR-2	Firmicutes	1	77
HP-GH10-70	*Sphingobacterium* sp. HP455	Bacteroidetes	1	98
HP-GH10-9	uncultured bacterium	-	1	75
HP-GH10-43	uncultured bacterium	-	15	65
HP-GH10-86	uncultured bacterium	-	16	75
HP-GH10-92	uncultured bacterium	-	1	67

Table S6. The group of GH 11 representative sequences identities.

Sequences	Closest Relative	Phylum	Number of Sequences	Identity %
HP-GH11-27	*Aspergillus fumigatus Af293*	Ascomycota	1	51
HP-GH11-34	*Aspergillus fumigatus Af293*	Ascomycota	1	52
HP-GH11-7	*Aspergillus fumigatus*	Ascomycota	17	53
HP-GH11-21	*Clostridium cellulolyticum H10*	Firmicutes	1	67
HP-GH11-66	*Clostridium papyrosolvens*	Firmicutes	1	64
HP-GH11-38	*Clostridium papyrosolvens*	Firmicutes	1	85
HP-GH11-16	*Dysgonomonas gadei*	Bacteroidetes	24	99
HP-GH11-17	*Dysgonomonas gadei*	Bacteroidetes	35	97
HP-GH11-19	*Dysgonomonas gadei*	Bacteroidetes	1	83
HP-GH11-89	*Dysgonomonas gadei*	Bacteroidetes	3	87
HP-GH11-35	*Dysgonomonas gadei*	Bacteroidetes	24	89
HP-GH11-1	*Dysgonomonas gadei*	Bacteroidetes	26	85
HP-GH11-29	*Paenibacillus curdlanolyticus*	Firmicutes	24	74
HP-GH11-51	*Paenibacillus* sp. Aloe-11	Firmicutes	1	90

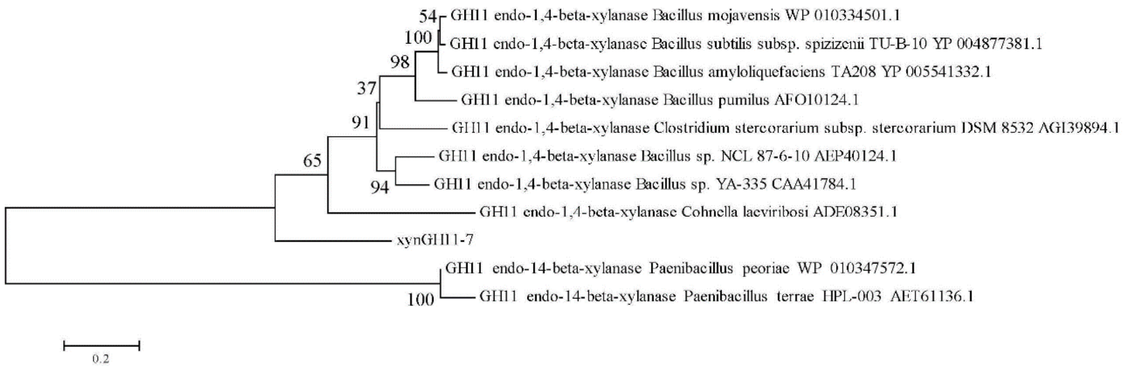

Figure S1. Phylogenetic tree constructed using the neighbor-joining method based on the endo-xylanase (xynGH11-7) sequence and the sequences of representative strains from GenBank. Bootstrap values (n = 1000 replicates) are reported as percentages. The scale bar indicates the number of changes per nucleotide position.
